# Purinergic Regulation of Neutrophil Function

**DOI:** 10.3389/fimmu.2018.00399

**Published:** 2018-03-01

**Authors:** Xu Wang, Deyu Chen

**Affiliations:** ^1^Institute of Oncology, Affiliated Hospital of Jiangsu University, Zhenjiang, China

**Keywords:** purinergic signaling, neutrophil, innate immune, inflammation, purinergic receptor

## Abstract

Purinergic signaling, which utilizes nucleotides (particularly ATP) and adenosine as transmitter molecules, plays an essential role in immune system. In the extracellular compartment, ATP predominantly functions as a pro-inflammatory molecule through activation of P2 receptors, whereas adenosine mostly functions as an anti-inflammatory molecule through activation of P1 receptors. Neutrophils are the most abundant immune cells in circulation and have emerged as an important component in orchestrating a complex series of events during inflammation. However, because of the destructive nature of neutrophil-derived inflammatory agents, neutrophil activation is fine-tuned, and purinergic signaling is intimately involved in this process. Indeed, shifting the balance between P2 and P1 signaling is critical for neutrophils to appropriately exert their immunologic activity. Here, we review the role of purinergic signaling in regulating neutrophil function, and discuss the potential of targeting purinergic signaling for the treatment of neutrophil-associated infectious and inflammatory diseases.

## Introduction

Purinergic signaling is among the most primitive signal transduction systems in evolutionary history (1). In humans, purinergic receptors (P2 and P1 receptors) are expressed in virtually all tissues and cell types, and they mediate a wide range of physiological and pathophysiological responses, such as neurotransmission, hypertension, inflammation, and cancer (2). Nucleotides (particularly ATP) and nucleosides (ADO), the basic elements of all living organisms, are well known for their function in energy metabolism. Notably, in the extracellular compartment, ATP and ADO are critical signal transduction molecules that participate in a wealth of different cellular responses through the activation of P2Rs and P1Rs, respectively (3).

The immune system is a tightly regulated and integrated cellular network that functions to preserve and restore homeostasis. The purinergic signaling system is an evolutionarily selected system that fine-tunes immune cell functions (4). Indeed, P2R- and P1R-mediated purinergic signaling frequently shows opposing effects in terms of modulating immune cell functions (5). Specifically, ATP-mediated P2 receptor signaling prevalently facilitates immune cell activation, whereas ADO-mediated P1R signaling mostly restricts immune cell activation (2). Shifting the balance from pro-inflammatory P2R signaling to anti-inflammatory P1R signaling or *vice versa* may have important consequences on the immune response outcome (3). Neutrophils are the most abundant immune cells in human blood and have emerged as an important component in orchestrating a complex series of events during inflammation (6). However, because of their short lifespan and how difficult they are to maintain in *in vitro* culture and to genetically manipulate, neutrophils are largely ignored in the purinergic signaling field. Recently, a wealth of pharmacologic and genetic evidence has shifted this paradigm by extending the role of purinergic signaling in neutrophils (7–9). Indeed, the coordinated interplay between P2 and P1 purinergic signaling is critical for neutrophils to effectively initiate their immunologic activity and restore tissue homeostasis. Here, we review the mounting evidence of neutrophil purinergic signaling and highlight their therapeutic potential in the treatment of neutrophil-associated infectious and inflammatory diseases.

## Extracellular ATP and ADO

In mammalian cells, ATP is synthesized by glycolysis or oxidative phosphorylation and stored at a high intracellular concentration of ~5 mM. In the physiological state, extracellular ATP is present in very minute amounts of ~10 nM due to plasma membrane-anchored ectonucleotidases (10). However, in pathological conditions, extracellular ATP concentrations are dramatically increased. Using a plasma membrane luciferase (pmeLUC) probe to monitor ATP levels *in situ*, investigators have demonstrated that the extracellular ATP level is nearly undetectable in healthy tissues, whereas in pathological conditions, such as graft-versus-host disease, acute hepatitis, and even primary tumor or metastases, the extracellular ATP concentration can be as high as a few hundred micromolar (11).

**Figure 1:**
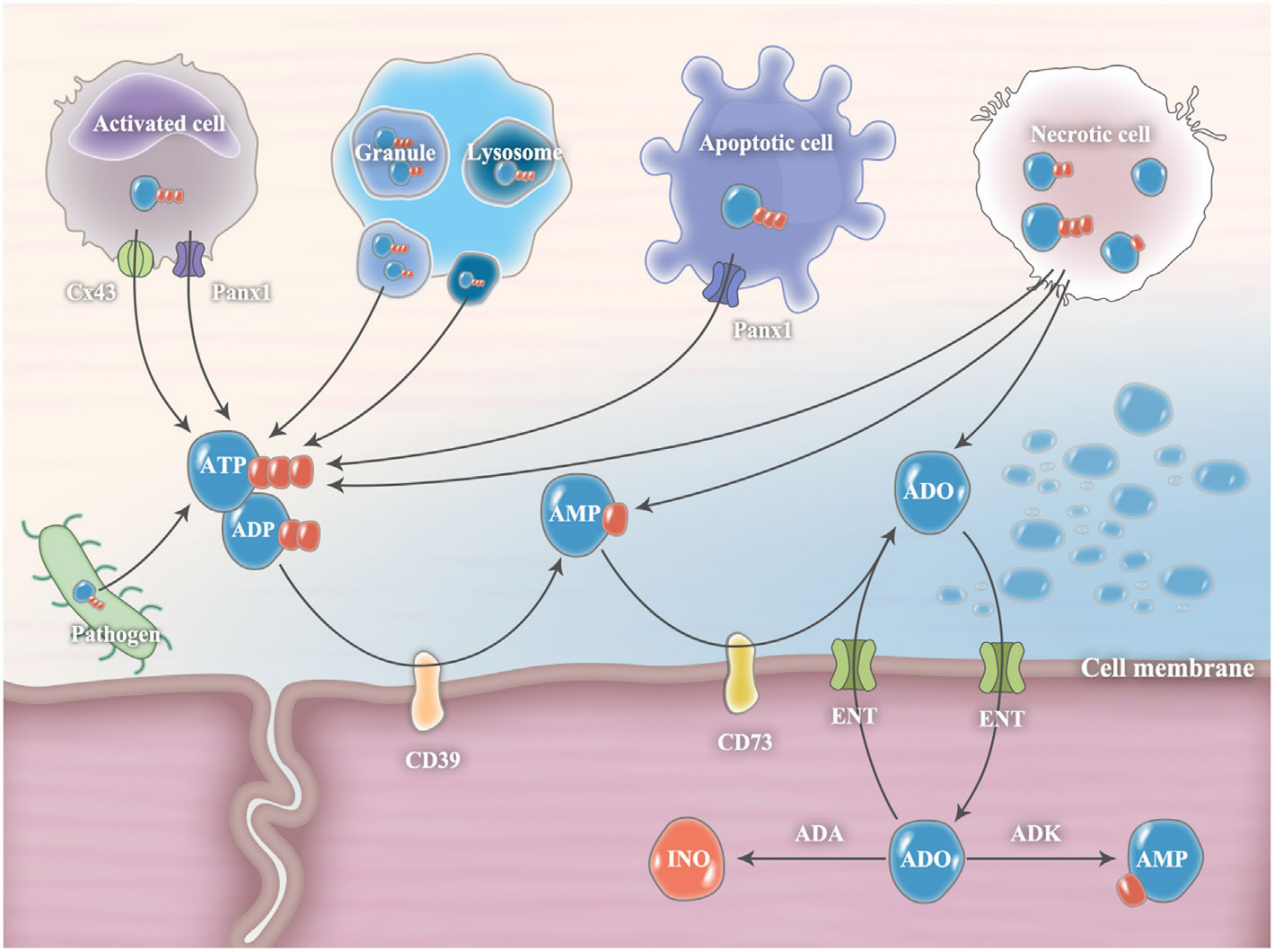
Release and metabolism of extracellular ATP and ADO. In pathological conditions, extracellular ATP release is finely controlled by diffusion through connexin 43 (Cx43) or pannexin 1 (Panx1) hemichannels and exocytotic release from ATP-rich vesicles (such as granule and lysosome). During cell apoptosis, Panx1 is cleaved by executioner enzymes of apoptosis (caspase 3 and 7) to generate a truncated and activated subunit that regulates ATP release from apoptotic cells. Under extreme conditions, cellular necrosis will release large amounts of ATP from intracellular storage pools. Pathogenic microorganisms, including bacteria and fungi, are also important sources of extracellular ATP. Extracellular ADP can be released from ADP-rich granules or necrotic cells. In the extracellular milieu, ATP and ADP levels are tightly controlled by plasma membrane ectonucleotidases, CD39 and CD73, which convert ATP/ADP to AMP and subsequently convert AMP to ADO, respectively. The accumulated ADO will be gradually transferred from the extracellular compartment into the intracellular compartment through equilibrative nucleoside transporters (ENTs) and subsequently metabolized to inosine (INO) by adenosine deaminase (ADA) or to AMP by adenosine kinase (ADK).

Under extreme conditions, such as trauma, ischemia, and infection, cellular necrosis will release large amounts of ATP from intracellular storage pools (Figure 1) (3). However, in most cases, extracellular ATP release is finely controlled by diffusion through plasmalemmal channels or exocytotic release from ATP-rich vesicles (2). Connexins and pannexin hemichannels are widely expressed throughout various cell types, including inflammatory cells, endothelial cells, and epithelial cells (12). Among them, connexin 43 (Cx43) and pannexin 1 (Panx1) hemichannels are the most studied. They were originally recognized as gap junction proteins that form non-covalent linkages between two cells and mediate cell-to-cell communications (12). However, isolated hemichannels can function as plasmalemmal channels between the cytoplasm and the extracellular space, thereby controlling ATP release (12). During cell apoptosis, Panx1 is cleaved by executioner enzymes of apoptosis (caspase 3 and 7) to generate a truncated and activated subunit that regulates ATP release from apoptotic cells (13). Recent evidence has demonstrated that Cx43 and Panx1 hemichannels are expressed in neutrophils and associated with the autocrine purinergic signaling that regulates neutrophil chemotaxis (8, 14). In addition, the exocytotic release of ATP from secretory vesicles (SVs) that specifically accumulate and store ATP is an important source of extracellular ATP. For example, dense granules in platelets contain large amounts of ATP (15). Once activated, ATP is readily secreted from platelets through exocytosis to mediate its biological functions in both autocrine and paracrine manners (15). Additional evidence shows that lysosomes are ATP-rich vesicles that can act as an important source of extracellular ATP through lysosomal exocytosis (16). Notably, pathogenic microorganisms, including bacteria and fungi, can release extracellular ATP (17). However, their roles in mediating host purinergic signaling have not been clarified.

Intracellularly, ADO is generated from hydrolysis of AMP by 5-nucleotidase or by hydrolysis of S-adenosyl-homocysteine (SAH) by SAH hydrolase (18). The intracellular adenosine concentration is quite variable. Minor changes in steady-state ATP levels (~5 mM) translate into major changes in intracellular adenosine concentrations (19). In the physiological state, extracellular ADO (~10 nM) is released constitutively from multiple cell types because of the ubiquitous presence of equilibrative nucleoside transporters (ENTs) (20). From the basal level, the extracellular ADO concentration can increase substantially (~100 times higher) in pathological conditions. The sources of extracellular adenosine are either from cell necrosis, equilibrative transporters, or ATP/ADP/AMP hydrolysis by nucleotidases (21). The accumulated ADO will be gradually transferred from the extracellular compartment into the intracellular compartment through ENTs and subsequently metabolized to INO by ADA or to AMP by adenosine kinase (ADK) (21).

In the extracellular milieu, ATP and ADP levels are tightly controlled by plasma membrane ectonucleotidases, such as nucleoside triphosphate diphosphohydrolase 1 (NTPDase1, also known as CD39) and ecto-5′-nucleotidase (also known as CD73), which convert ATP/ADP to AMP and subsequently convert AMP to ADO, respectively (5). The CD39/CD73 pathway is a critical checkpoint, driving a shift from an ATP-induced pro-inflammatory environment to an anti-inflammatory milieu induced by ADO (5). Both CD39 and CD73 are expressed in neutrophils and appear to be critical players in the regulation of neutrophil activation (7). In addition to CD39 and CD73, nucleotide pyrophosphatases and phosphodiesterases (NPPs), alkaline phosphatases (ALP), acid phosphatases (ACP), and ectokinases can also degrade extracellular nucleotides and nucleosides; however, their roles in regulating immune responses are less well understood (3).

## Purinergic Receptors

Separate families of receptors for ATP (P2 receptors) and ADO (P1 receptors) were first identified in 1978. According to their transduction mechanism and molecular cloning, P2 receptors are subsequently divided into ionotropic P2XRs and metabotropic P2YRs. At present, seven P2XRs (P2X_1–7_R), eight P2YRs (P2Y_1/2/4/6/11/12/13/14_R), and four P1 receptors (A_1_/A_2A_/A_2B_/A_3_R) have been cloned and characterized (2).

### P2XRs

Ionotropic P2XRs are ATP-gated trimeric ion channels (3). P2XRs share a similar tertiary topology with an intracellular NH_2_ and a longer COOH terminus, a large extracellular loop responsible for ligand binding, and two transmembrane-spanning regions (TM1 and TM2) (3). TM1 is involved in channel gating and a helix of TM2 forms channel pore (3). Following ATP binding, P2XRs become permeable to Na^+^, K^+^, and Ca^2+^. Moreover, activation of P2X_7_R forms a large pore that allows the passage of molecules as large as 900 Da, which is associated with the release of pro-inflammatory cytokines (22).

### P2YRs

P2YRs belong to the δ-branch of class A G-protein-coupled receptor (GPCR) family and contain seven hydrophobic transmembrane regions connected by three extracellular loops and three intracellular loops (3). Based on the phylogenetic and structural divergence, two distinct P2YR subgroups have been identified (23). The first group contains P2Y_1/2/4/6/11_Rs, with a sequence homology of 35–52% in amino-acid composition and the presence of a Y–Q/K–X–X–R defining motif in the transmembrane α-helix 7. The second group contains P2Y_12/13/14_Rs, with a sequence homology of 47–48% and the presence of the K–E–X–X–L motif in transmembrane α-helix 7. Moreover, the two subgroups also differ in their primary coupling to G-proteins (23). P2Y_1/2/4/6/11_Rs primarily couple to G_q_/G_11_ and initiate phospholipase C/inositol trisphosphate/diacylglycerol pathway to increase intracellular calcium, whereas P2Y_12/13/14_Rs principally couple to G_i/0_ and inhibit adenylate cyclase (AC) to decrease intracellular cyclic AMP (cAMP). Additional evidence shows that P2Y_11_R uses G_s_ to stimulate AC and increase intracellular cAMP.

### P1Rs

P1Rs belong to classical G-protein-coupled metabotropic receptors, and are single polypeptides comprising seven α-helices oriented perpendicular to the plasmalemma (24). There is a close similarity in sequence of A_1_R, A_2A_R, A_2B_R, and A_3_R. A_2A_R shares a sequence identity of 46, 37, or 31% with A_2B_R, A_1_R, and A_3_R, respectively (24). ADO is the endogenous ligand for all four ADO receptors. A_1_R and A_3_R inhibit adenylyl cyclase activity through G_i_ G-proteins, and stimulate phospholipase C/inositol trisphosphate/diacylglycerol pathway *via* Gβγ G-proteins (4). A_2A_R and A_2B_R primarily couple to G_s_ G-proteins to increase AC activity (4). Moreover, all four ADO receptors couple to mitogen-activated protein kinases (MAPKs), which renders them more sophisticated biological functions (4).

### Expression of Purinergic Receptors in Neutrophils

In neutrophils, expression analyses have been performed for all purinergic receptors, except P2Y_12_R (Table 1). Convincing data obtained from mRNA, protein, and functional assays have demonstrated that P2X_1_R, P2X_7_R, P2Y_2_R, P2Y_14_R, and all four ADO receptors are expressed in neutrophils. Expression evidence for other purinergic receptors is relatively weak, and additional work is needed to further confirm whether P2X_2-6_Rs, P2Y_1_R, P2Y_4_R, P2Y_6_R, P2Y_11_R, P2Y_12_R, and P2Y_13_R are expressed in neutrophils.

**Table 1:** Expression and/or function of purinergic receptors in neutrophils.

Preferred ligands	Receptor	Expression			Regulation of neutrophil functions	Reference
		Species	Detected	Evidence		
ATP	P2X_1_R	Human	+	R, P, F	Chemokinesis ↑	(8, 35)
			–	P, F	Transmigration ↑	
		Mouse	+	P	Phagocytosis ↑	
		Rat	+	R	Degranulation ↑	
					Chemotaxis ↓	

ATP	P2X_2_R	Human	–	R	No information available	
		Rat	–	R		

ATP	P2X_3_R	Human	–	R	No information available	
		Rat	–	R		

ATP	P2X_4_R	Human	–	R	No information available	
		Rat	+	R		

ATP	P2X_5_R	Human	+	R	No information available	
		Rat	+	R		

ATP	P2X_6_R	Human	–	R	No information available	
		Rat	–	R		

ATP	P2X_7_R	Human	+	R, P, F	IL-1β secretion ↑	(9)
			–	R, P, F		
		Mouse	+	R, P, F		
			–	R		
		Rat	+	R		

ADP	P2Y_1_R	Human	–	R	No information available	

ATP = UTP	P2Y_2_R	Human	+	R, P, F	Chemotaxis ↑Oxidative burst ↑	(7, 28, 43)
			–	R		
		Mouse	+	R		
		Rat	+	P		

UTP	P2Y_4_R	Human	+	R	No information available	

UDP	P2Y_6_R	Human	+	R, P	NET formation ↑	(40)
		Rat	+	P		

ATP	P2Y_11_R	Human	+	R, P	Chemotaxis ↑Apoptosis↓	(31, 49)
		Rat	+	P		

ADP	P2Y_12_R	No information available				

ADP	P2Y_13_R	Mice	+	P	No information available	

UDP glucose	P2Y_14_R	Human	+	R, F	Chemotaxis ↑	(32)
		Mice	+	P		

ADO	A_1_R	Human	+	R, P, F	Oxidative burst ↓Chemotaxis ↑	(30)
			–	P		
		Mouse	–	P		

ADO	A_2A_R	Human	+	R, P, F	Rolling and adhesion ↓Chemotaxis ↑	(29, 36)
		Mouse	+	R, F		
		Horse	+	F		

ADO	A_2B_R	Human	+	R, P, F	NET formation ↓Oxidative burst ↓	(41)
			–	R		
		Mouse	+	R, P		

ADO	A_3_R	Human	+	R, P, F	Oxidative burst ↓Phagocytosis ↑Chemotaxis ↑	(7, 42)
		Mouse	+	R, F		
		Rat	+	P		

*R, mRNA; P, protein; F, function; NET, neutrophil extracellular trap. Function evidence includes electrophysiological study, calcium imaging, and radioligand binding assay*.

## Regulation of Neutrophil Function by Purinergic Receptors

### Purinergic Regulation of Neutrophil Chemotaxis

Chemotaxis denotes the ability of cells to sense gradients, polarize, and directionally migrate within a chemotactic gradient field. Recent studies have highlighted the critical role of autocrine purinergic signaling in directing neutrophil chemotaxis (Table 1) (25). During chemotaxis, neutrophils require polarization, with an anterior pseudopod at the leading edge to sense chemoattractant gradients and a posterior pseudopod at the trailing edge to maintain orientated migration (26). Once neutrophils sense the chemoattractants, Panx1 hemichannels rapidly translocate to the leading edge and release mitochondria-derived ATP from pseudopod protrusions (27). The extracellular release of ATP serves as an autocrine messenger to amplify chemotaxis signals through activation of P2Y_2_R-mediated mTOR signaling at the leading edge (28). Extracellular ATP and positive feedback *via* the P2Y_2_R receptor constitutes a mechanism that is required for neutrophil gradient sensing (7). Then, released ATP is hydrolyzed to ADO *in situ* by neutrophil membrane-associated CD73, which subsequently activates neutrophil A_3_R at the leading edge to drive a second round of signal amplification (7). The second amplification step is equally important to the initial amplification that facilitates neutrophil chemotaxis because it controls migration speed (7). At the trailing edge, diffused or locally generated ADO activates A_2A_R and triggers cAMP/PKA signaling to inhibit P2Y_2_R-mediated mTOR signaling (29). The activation of A_2A_R maintains cell polarization and promotes uropod retraction. In polarized neutrophils, the P2Y_2_R receptor remains uniformly distributed across the cell membrane, whereas CD73 and A_3_R accumulate at the leading edge, and A_2A_R translocates to the trailing edge.

The described autocrine purinergic signaling axis plays crucial roles in mediating neutrophil chemotaxis in a chemotactic gradient field. In addition to P2Y_2_R, A_3_R, and A_2A_R, other purinergic receptors also mediate neutrophil chemotaxis. Activation of P2X_1_R by extracellular ATP fails to induce neutrophil directional chemotaxis, but it can enhance neutrophil chemokinesis (random cell migration) through Rho kinase signaling (8). Moreover, the lipopolysaccharide (LPS)-induced autocrine release of ATP inhibits neutrophil chemotaxis in a chemotactic gradient field *via* P2X_1_R (8). LPS activates neutrophil Cx43 hemichannels to release extracellular ATP, which binds to P2X_1_R and initiates Ca^2+^ influx. The Ca^2+^ influx subsequently inhibits neutrophil chemotaxis by enhancing myosin light chain phosphorylation, which interferes with neutrophil polarization. Knockout of P2X_1_R reverses LPS-inhibited neutrophil chemotaxis. In contrast to P2X_1_R, A_1_R facilitates neutrophil chemotaxis in the presence of LPS (30). ADO and an agonist to A_1_R can restore neutrophil chemotaxis by inhibiting the LPS-induced p38 kinase. Additional studies indicate that neutrophil P2Y_11_R and P2Y_14_R receptors also enhance neutrophil chemotaxis, but the mechanisms are not well understood (31, 32).

### Purinergic Regulation of Neutrophil Rolling, Adhesion, and Transmigration

Neutrophils are typically the first immune cells that are recruited to inflammatory sites (33). In most tissues, the neutrophil recruitment cascade involves the following commonly recognized steps: tethering, rolling, adhesion, crawling, and subsequent transmigration (34). A recent *in vivo* study demonstrated that LPS-induced neutrophil rolling and adhesion in cremaster muscle postcapillary venules does not differ between WT and *P2rx1^−/−^* mice (35). However, neutrophil transmigration is inhibited in *P2rx1^−/−^* mice, indicating that P2 × 1 signaling may participate in the neutrophil recruitment cascade by promoting neutrophil transmigration but not rolling and adhesion (Table 1). Using adoptive transfer of neutrophils from WT and *P2rx1^−/−^* mice into WT mice, the results demonstrated that the absence of the P2X_1_R in neutrophils, but not in vascular endothelial cells or other immune cells, is responsible for neutrophil emigration from venules. In contrast to P2X_1_R, neutrophil-expressed A_2A_R appears to inhibit the neutrophil recruitment cascade. Using an A_2A_R agonist to initiate A_2A_R signaling, β2 integrin-mediated neutrophil rolling and adhesion are markedly inhibited both in TNF-α-challenged murine cremaster muscle postcapillary venules and in *ex vivo* flow chamber models (36).

### Purinergic Regulation of Neutrophil Phagocytosis and Neutrophil Extracellular Traps (NETs)

Neutrophils are professional phagocytes that are endowed with a unique capacity to engulf and thereby eliminate pathogens and cell debris. The discovery of NETs has extended the understanding of neutrophil antimicrobial strategies (37). NETs are large, extracellular, web-like structures that are composed of decondensed chromatin and neutrophil antimicrobial factors. NETs can not only trap and kill a variety of microbes but also activate and regulate innate and adaptive immunity (38). Recent studies identified a cell-autonomous, microbe size-sensing mechanism that allows neutrophils to sense pathogen size and respond by phagocytosing small pathogens or selectively releasing NETs in response to large pathogens (39). Phagocytosis and NETs are both crucial for neutrophils to efficiently eliminate invading pathogens.

As a potent activator of neutrophils, LPS enhances neutrophil phagocytosis of *Escherichia coli* (*E. coli*) in humans (8). However, the promotive effects of LPS are abolished when a P2X_1_R antagonist is introduced (8). Because LPS induces neutrophils to release extracellular ATP, these results suggest that autocrine activation of P2 × 1 signaling may be essential for enhancing neutrophil phagocytosis (Table 1) (8). UDP is the natural ligand to P2Y_6_R. While UDP itself fails to initiate NET formation in human neutrophils, UDP-mediated P2Y6 signaling is involved in the monosodium urate crystal-induced formation of NETs (40). When incubated with *Klebsiella pneumoniae*, neutrophil A_2B_R expression is increased by approximately 500-fold (41). In addition, neutrophils from *Adora2b^−/−^* mice show a greatly enhanced ability to kill bacteria compared with that of neutrophils from WT mice. The following studies demonstrate that A_2B_R impairs the neutrophils’ ability to kill *Klebsiella pneumoniae* by suppressing NET formation but not phagocytosis. Neutrophil A_3_R enhances bacterial clearance, and activating human neutrophils with an A_3_R agonist promotes the formation of neutrophil filipodia-like projections, which are named cytonemes (42). The formation of these structures enables neutrophils to sample, capture and “reel in” pathogens to induce phagocytosis.

### Purinergic Regulation of Neutrophil Oxidative Burst

Superoxide serves as a potent antimicrobial agent to kill microbial pathogens and modulates multiple signaling pathways. Because of the destructive nature of superoxide, the oxidative burst is fine-tuned, and purinergic signaling is intimately involved in this process (Table 1). P2Y_2_R is an activator of neutrophil oxidative burst (43). Knockdown of P2Y_2_R in differentiated neutrophil-like HL-60 cells (dHL-60) significantly inhibits fMLP-induced oxidative burst. Monosodium urate crystals can induce neutrophil oxidative burst, but an antagonist to P2Y_6_R suppresses this monosodium urate crystal-induced neutrophil oxidative burst (40). Notably, exogenous ATP was not administered in these two studies, and autocrine activation of neutrophil P2Y_2_R and P2Y_6_R may amplify the oxidative burst. In contrast to P2Y_2_R and P2Y_6_R, A_2B_R, and A_3_R inhibit the neutrophil oxidative burst. Agonists to A_2B_R or A_3_R significantly inhibit stimulus-induced superoxide production in WT neutrophils but not in *Adora2b^−/−^* or *Adora3^−/−^* neutrophils (44, 45).

### Purinergic Regulation of Neutrophil Degranulation

Neutrophil granules, including primary granules (PGs), secondary granules (SGs), tertiary granules (TGs), and SVs, are formed sequentially during granulopoiesis (46). Neutrophil granules contain a multitude of antimicrobial peptides and proteolytic enzymes. These proteins enable neutrophils to exert their bactericidal and immunologic functions but are potentially harmful to the host if released inappropriately. Purinergic signaling plays bidirectional roles in regulating neutrophil degranulation (Table 1). An *in vitro* study showed that fMLP-induced neutrophil degranulation can be further enhanced by a non-hydrolyzable ATP analog, ATPγS, but not by hydrolyzable ATP (47). Instead, hydrolyzable ATP suppresses fMLP-induced neutrophil degranulation. Given the potent hydrolytic activity of neutrophil membrane ectonucleotidases, which converts ATP to ADO, the following studies reveal that the inhibitory effect on neutrophil degranulation is induced by the hydrolysis products of ATP, ADO. Furthermore, with the application of selective agonists and antagonists, a recent study indicated that LPS-induced autocrine release of ATP promotes neutrophil exocytosis of SVs, TGs, and SGs *via* activation of P2X_1_R (8). The described bidirectional effects of purinergic signaling on neutrophil degranulation may be required for neutrophils to appropriately release their granule contents to regulate their antimicrobial activity during infection and avoid damaging healthy tissues.

### Purinergic Regulation of Neutrophil Apoptosis

Circulating neutrophils have a very short lifespan of 8–20 h and do not proliferate (48). However, under inflammation and other pathologic states, neutrophil lifespan is markedly prolonged (48). Extracellular ATP is a critical regulator that inhibits neutrophil apoptosis. Even a 10-min exposure to ATP is sufficient to cause a sustained delay of neutrophil apoptosis in humans (49). Using various selective purinergic receptor antagonists, investigators have identified that ATP-mediated delay of neutrophil apoptosis is P2Y_11_R-dependent (49). P2Y_11_R mediates the anti-apoptotic effect of ATP through elevation of neutrophil intracellular cAMP and activation of the subsequent cAMP-dependent protein kinases.

## Purinergic Signaling Shapes Neutrophil Immunity in Pathological Conditions

### Bacterial Infection and Sterile Inflammation

Neutrophils are the first line of defense within the immune system, and these cells infiltrate diseased tissues to eliminate invading pathogens and necrotic debris, with the aim of restoring tissue homeostasis (50). During *Streptococcus pneumoniae* corneal infection in mice, neutrophils are the predominant P2X_7_R-expressing inflammatory cells to infiltrate the infection (9). Knockout of P2X_7_R has no influence on neutrophil infiltration into the infected cornea but significantly impairs bacterial clearance. The following adoptive transfer experiments using WT or *P2rx7^−/−^* neutrophils indicated that P2X_7_R-expressing neutrophils are required for bacterial clearance. Neutrophil P2X_7_R exacerbates the local immune responses by mediating ATP-induced NLRP3 inflammasome activation and IL-1β secretion. In contrast to P2X_7_R, neutrophil A_2B_R impairs antimicrobial activity during *Klebsiella pneumoniae* infection (41). *Adora2b^−/−^* mice demonstrate enhanced bacterial clearance but unaltered neutrophil infiltration in infected lungs, compared with WT mice. Furthermore, WT recipients of *Adora2b^−/−^* BM show a comparable degree of protection compared with global *Adora2b^−/−^* knockout mice. The enhanced bactericidal effects appear to be associated with neutrophils, because neutrophils from *Adora2b^−/−^* mice demonstrate enhanced extracellular bactericidal activity *via* generation of NETs.

In thermal injury-induced mice with sterile liver inflammation, selective inhibition or knockout of P2X_7_R results in reduced hepatic neutrophil recruitment (51). BM chimeric mice demonstrate that P2X_7_R targets cells that are of hematopoietic origin but are not neutrophils because isolated *P2rx7^−/−^* neutrophils are recruited equivalently to inflammatory foci after adoptive transfer. Significant ATP release and increased expression of P2Y_2_R in mice liver are observed during concanavalin A-mediated hepatitis (52). Selective inhibition or knockout of P2Y_2_R protects against hepatitis and neutrophil infiltration. Chimeric mice demonstrate that P2Y_2_R is required in BM-derived cells for hepatic neutrophil infiltration and subsequent liver damage. Inhibition of P2X_3_R and P2X_2/3_R with a selective antagonist reduces rat neutrophil infiltration into stimulus-induced inflamed knee joints (53) but not skin and subcutaneous tissues (54, 55). Similarly, P2X_7_R antagonism inhibits rat neutrophil infiltration into inflamed knee joints (56), but knockout of P2X_7_R has no effect on skin neutrophil infiltration (57). The variations between neutrophil infiltration in joints and skin tissues are probably caused by specific chemoattractants and the order of their action during neutrophil recruitment at different tissues (58).

### Acute Lung Injury (ALI)

Acute lung injury is characterized by lung edema, endothelial and epithelial injury and an excessive infiltration of neutrophils into the interstitium and bronchoalveolar space (59). Neutrophils play a key role in the progression of ALI (60). Neutrophils infiltrate lung tissues, occlude pulmonary capillaries, release cytotoxic mediators such as proteolytic enzymes and superoxide, and thereby destroy lung tissues.

Intratracheal administration of LPS is classically used to induce ALI. With this experimental model, several genetic and pharmacological studies have confirmed that inappropriate activation of P2R signaling is associated with neutrophil-induced hyperinflammation and tissue damage. Knockout or antagonism of mouse P2X_7_R inhibits neutrophil recruitment into the lung and protects against ALI (61–63). LPS provides a dual signal for alveolar macrophages and induces cytokine production *via* TLR4/MyD88 signaling and necrosis *via* P2X_7_R/CD14 signaling, which promotes the release of pro-IL-1α and subsequently activates the IL-1 receptor on endothelial cells to induce tight junction opening and allow neutrophil infiltration into the lungs (62). In addition, the P2X_7_R-induced shedding of soluble VCAM-1 from alveolar epithelial type-I cells functions as a chemoattractant to recruit neutrophils during ALI (63). The pulmonary neutrophil recruitment induced by intratracheal LPS can also be inhibited in mice that receive either with P2Y_1_R or P2Y_14_R antagonists (64). Activation of P2Y_1_R and P2Y_14_R in platelets induces a platelet-dependent neutrophil motility toward macrophage-associated chemokines. In contrast to P2R signaling, activation of P1R signaling frequently interferes with neutrophil trafficking during ALI. During exposure to LPS, mouse lung A_1_R expression is upregulated (65). However, when neutrophils are depleted before LPS inhalation, the induction of A_1_R is attenuated, suggesting that neutrophils induce A_1_R in the inflamed lungs (65). Moreover, LPS-induced transmigration of neutrophils into the interstitium and bronchoalveolar lavage is further elevated in *Adora1^−/−^* mice but can be reversed by an A_1_R agonist in WT mice. These findings were further confirmed by *in vitro* experiments showing that chemokine-induced transmigration is reduced when neutrophils are pretreated with an A_1_R agonist and that an A_1_R agonist can prevent pulmonary endothelial cells from LPS-induced cellular remodeling and cell retraction. Lung A_2A_R expression is increased when mice are exposed to intratracheal LPS (66). LPS-induced neutrophil accumulation in the pulmonary circulation, but not interstitium and bronchoalveolar lavage, is significantly greater in *Adoa2a^−/−^* mice than in WT mice (66). In chimeric mice lacking A_2A_R on BM-derived cells, neutrophil migration into the bronchoalveolar lavage is increased, and an A_2A_R agonist reduces LPS-induced neutrophil recruitment only when A_2A_R is expressed on BM-derived cells (66, 67). Similar results are observed with mice that selectively lack A_2A_R on myeloid cells; these mice show increased migration of neutrophils into the bronchoalveolar lavage (66). Taken together, these data indicate that A_2A_R activation on BM-derived cells is critical for pulmonary neutrophil infiltration during ALI. Intratracheal administration of LPS induces A_2B_R expression in the lung, and *Adoa2b^−/−^* mice show an increase in neutrophil migration into the pulmonary interstitium (68). Using chimeric mice, investigators identified that A_2B_R on BM-derived cells is crucial for inhibiting neutrophil migration during ALI (68, 69). However, in a two-hit ALI model where intratracheal LPS treatment is followed by injurious mechanical ventilation, tissue-specific *Adora2b^−/−^* in alveolar epithelial cells, but not myeloid lineage or endothelial cells, closely resembles the observed detrimental effects of global *Adora2b^−/−^* (70). Differences in the pathological environments induced by mechanical ventilation may contribute to this discrepancy.

In severe pulmonary infection-induced ALI models, P2X_7_R is involved in the exacerbated inflammatory injuries and neutrophil infiltration. Severe tuberculosis and pulmonary injury are caused when mice are infected with hypervirulent *Mycobacterium bovis* (71). Chimeric mice that lack P2X_7_R in BM-derived cells show alleviated pulmonary injury, which demonstrates that P2X_7_R in BM-derived cells plays a critical role in the progression of severe tuberculosis. An exacerbated immune response is one of the main causes of influenza virus-induced lung damage during infection (72). Knockout of P2X_7_R results in a better outcome in response to influenza virus infection, as characterized by reduced lung pathology and neutrophil infiltration. Moreover, the absence of P2X_7_R or inhibition of P2X_7_R activation by selective antagonist or apyrase suppresses pulmonary inflammatory responses and neutrophil infiltration in the early phase of acute adenoviral infection (73).

Acute lung injury represents the primary complication in sepsis during the sequential development of multiple organ dysfunction (74). In intraperitoneal LPS injection-induced SIRS-associated ALI, P2X_1_R mediates pulmonary neutrophil infiltration. Lecut et al. reported that a deficiency of P2X_1_R augments neutrophil accumulation in the lungs and tissue damage (75), whereas Maître et al. observed that deficiency of P2X_1_R leads to strongly reduced neutrophil accumulation in the lungs and less tissue damage (76). The differences could be related to the experimental design (e.g., monitoring of age, sex, and weight matching) or to the dose and serotype of LPS. Further investigations will be necessary to confirm these findings (76). Expression of lung A_2A_R is upregulated in mice that are intravenously injected with LPS (77). LPS-induced accumulation of pulmonary neutrophils is further enhanced in *Adora2a^−/−^* mice but can be reversed in WT mice by an A_2A_R agonist, indicating the protective effects of A_2A_R against ALI. Cecal ligation and puncture (CLP) is an alternative approach to induce SIRS-associated ALI. Performing CLP in *P2ry2^−/−^* and *Adora3^−/−^* mice, investigators observed decreased recruitment of neutrophils into the lungs and attenuated development of ALI compared with WT mice (78). Similar results were observed in experiments using *P2ry12^−/−^*mice, and antagonists of P2Y_12_R succeeded in reversing the pathological changes in WT mice (79, 80). These studies demonstrate the stimulatory role of P2Y_2_R, A_3_R, and P2Y_12_R in neutrophil infiltration during ALI.

### Ischemia-Reperfusion (IR) Injury

Ischemia-reperfusion injury occurs when blood and concomitant oxygen return to tissues after the initial insult of ischemia or lack of oxygen, which is a common complication following myocardial infarction, transplantation, stroke and trauma (81). Restoration of circulation and reoxygenation are frequently associated with inflammatory responses and subsequent tissue injuries, rather than recovery of normal tissue functions (81). IR injury is recognized as a complex cascade of events, involving numerous biochemical compounds that are released in response to ischemia and interactions between vascular endothelial cells and immune cells (81). Neutrophil infiltration is a hallmark of IR injury and represents an important component in the protracted inflammatory response and its severity (82). The profound effects of purinergic signaling on neutrophils during IR injury have been identified.

Convincing evidence obtained from *Adora1^−/−^* mice demonstrates that A_1_R signaling is essential for protecting against multiple IR-induced organ injuries (83–85). *Adora1^−/−^* mice exhibit significantly higher neutrophil infiltration and tissue inflammatory responses during IR-induced renal, pulmonary and hepatic injuries than WT mice (83–85). In addition, A_1_R agonists can maintain the neutrophil infiltration and tissue inflammatory responses in WT mice but not in *Adora1^−/−^* mice (84, 85). In parallel with the A_1_R agonist, an A_1_R allosteric enhancer that selectively increases the efficacy of endogenous ADO–A_1_R interaction protects against neutrophil infiltration and renal IR injury in WT mice but not in *Adora1^−/−^* mice (83). Suppressed neutrophil infiltration and the accompanying beneficial effects have been achieved during renal, pulmonary and myocardial IR injuries by administration of A_2A_R agonists in various experimental animals (86–88). Nevertheless, when the agonist is applied in *Adora2a^−/−^* mice or paired with a specific antagonist, suppression of neutrophil infiltration and all the protective effects are eliminated (88, 89). These findings reveal the roles of A_2A_R signaling in inhibiting neutrophil infiltration and protecting against tissue injuries during renal, pulmonary and myocardial IR. Agonism of A_3_R provides significant protection against neutrophil infiltration, lung inflammation and dysfunction after pulmonary IR injury in WT mice but not *Adora3^−/−^* mice (90). Further *in vitro* transwell assays have shown that an A_3_R agonist inhibits neutrophil transmigration, suggesting that the protective effects of A_3_R may be due to the direct prevention of neutrophil activation. In addition, A_3_R activation reduces neutrophil infiltration into IR-injured myocardium in WT mice but not in global A_3_R deficient mice or chimeric mice that lack of A_3_R in BM-derived cells (22). Subsequent experiments using *in vitro* transwell assays achieved results that are consistent with previous studies, indicating that neutrophil-expressed A_3_R is essential for inhibiting neutrophil infiltration.

In contrast to the effects of A_1_R, A_2A_R, and A_3_R for suppressing neutrophil activation during IR injury, A_2B_R exhibits a bidirectional role in myocardial and pulmonary IR injury. Evidence from chimeric mice that lack A_2B_R in BM cells shows that A_2B_R is protective when activated on BM-derived cells (most likely neutrophils) in myocardial IR injury (91). The following study that used mice with a specific deletion of A_2B_R in the myeloid lineage or either endothelial or myocardial cells demonstrated that myeloid lineage cells are necessary to provide cardioprotection during IR injury and inhibit neutrophil accumulation (92). Adoptive transfer of *Adora2b^−/−^* neutrophils into neutrophil-depleted mice further confirmed the role of neutrophil-expressed A_2B_R in cardioprotection against IR injury (92). In contrast to myocardial IR injury, A_2B_R appears to promote pulmonary IR injury and inflammatory responses by stimulating the production of cytokines and neutrophil infiltration (93, 94). However, the pro-inflammatory effects of A_2B_R are likely specific for resident pulmonary cells, but not BM-derived neutrophils, based on the data obtained from BM chimeric mice (94).

Compared with the considerable evidence showing that ADO-driven P1 signaling mediates neutrophil activation and infiltration during IR injury, few studies have focused on the effects of ATP-induced P2R signaling on neutrophil function during IR injury. A recent report demonstrated that antagonism of P2X_7_R ameliorates IR-induced renal neutrophil infiltration and tissue injury, and similar results were achieved by knockout of P2X_7_R (95). Chimeric mice with P2X_7_R deficiency in BM cells further confirm that activation of P2X_7_R in BM-derived cells is essential for renal neutrophil infiltration during IR injury. Additional studies are needed to further clarify the effects of other P2 receptors on neutrophils during IR injury.

## Concluding Remarks and Future Perspectives

**Figure 2:**
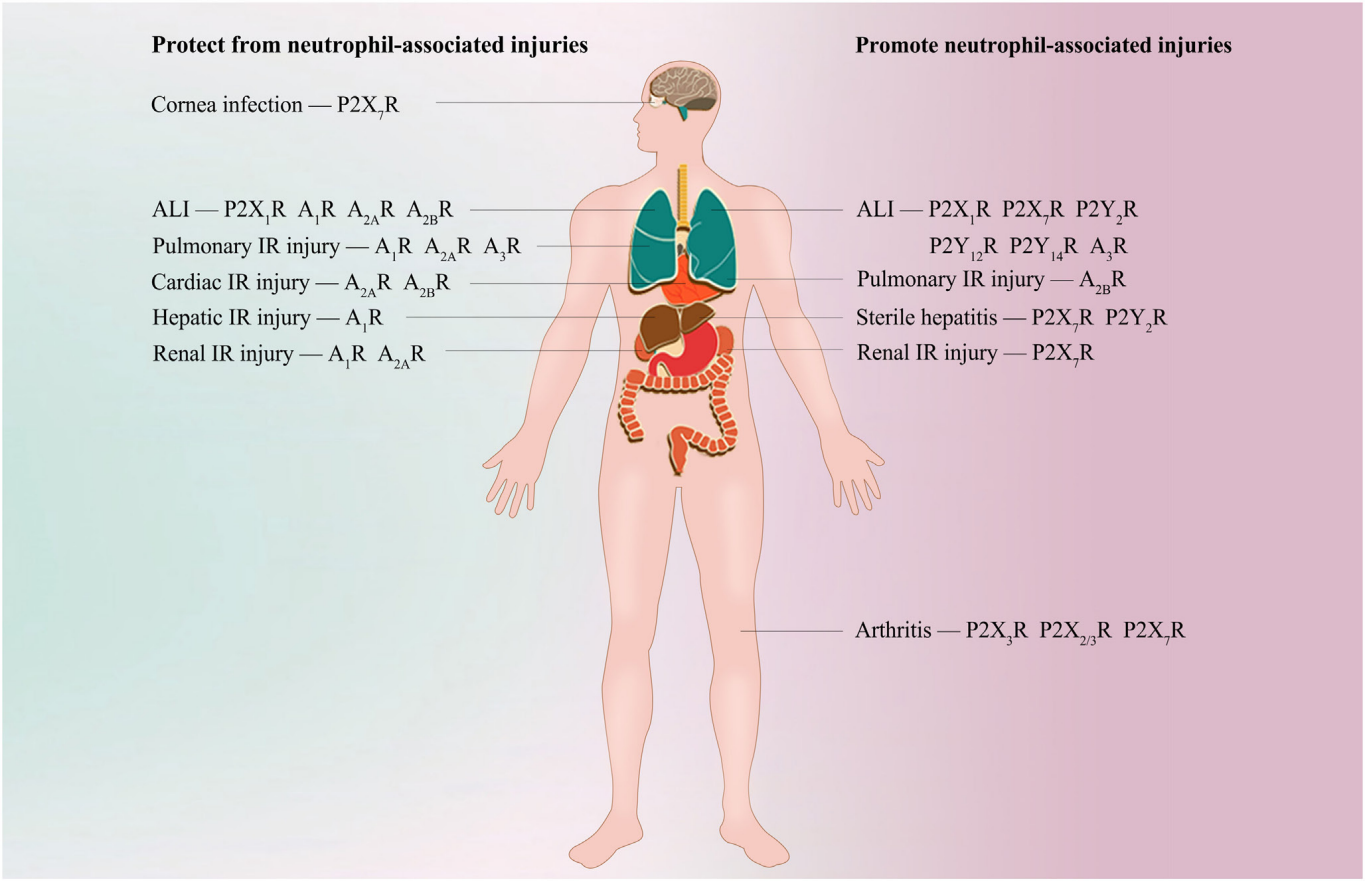
Potential purinergic targets for treating neutrophil-associated diseases. During bacterial clearance in *Streptococcus pneumoniae* corneal infection, agonism of neutrophil P2X_7_R is required for bacterial clearance. Inappropriate activation of P2X_7_R, P2Y_2_R, P2Y_12_R, P2Y_14_R, and A_3_R is associated with neutrophil-induced hyperinflammation and tissue damage during acute lung injury (ALI); however, A_1_R, A_2A_R, and A_2B_R are essential for protecting against ALI. Specific antagonism of P2Y_2_R, P2Y_12_R, P2Y_14_R, and A_3_R or specific agonism of A_1_R, A_2A_R, and A_2B_R alleviates neutrophil recruitment into the lung and protects against ALI. P2X_1_R exhibits a bidirectional role in neutrophil accumulation and tissue damage during ALI, but the detailed mechanisms are unclear. A_1_R, A_2A_R, and A_3_R signaling frequently inhibits neutrophil infiltration and protects against tissue injuries during pulmonary, cardiac, hepatic, or renal ischemia-reperfusion (IR) injury. However, evidence shows that A_2B_R is protective when activated on BM-derived cells (most likely neutrophils) in myocardial IR injury, but promote pulmonary IR injury when activated on resident pulmonary cells. During renal IR, antagonism of P2X_7_R ameliorates neutrophil infiltration and renal injury. P2X_7_R or P2Y_2_R is required for hepatic neutrophil infiltration and subsequent liver damage in thermal or chemical injury-induced sterile hepatitis, respectively. Specific inhibition of P2X_7_R or P2Y_2_R is shown to protect against neutrophil infiltration and liver inflammatory injuries. Antagonism of P2X_3_R, P2X_2/3_R, and P2X_7_R reduces neutrophil infiltration into inflamed knee joints in arthritis.

The past 5 years have brought major advances in the identification of numerous types of neutrophil purinergic receptors and in the understanding of their functions in coordinating the appropriate immune response against invading pathogens or diseased tissue. The fine-tuned balance between P2R and P1R signaling appears to be critical for shaping neutrophil plasticity and heterogeneity to orchestrate a complex series of events during inflammation (Figure 2). Several drugs that target purinergic signaling (such as adenosine and clopidogrel) have already used in patients. More selective and effective pharmacological tools are continually being developed, and increasing developments in the field of neutrophil purinergic signaling may be further exploited in the treatment of patients with inflammatory or infectious diseases in the near future. In keeping with this goal, key future challenges will be to understand the nature of the neutrophil purinergic signaling repertoire during *in vivo* infectious and inflammatory conditions, how purinergic receptors are integrated with other pattern recognition receptors to control neutrophil function and the dynamic biochemistry of ATP and ADO in the extracellular environment.

## Author Contributions

All authors listed have made a substantial, direct, and intellectual contribution to the work and approved it for publication.

## Conflict of Interest Statement

The authors declare that the research was conducted in the absence of any commercial or financial relationships that could be construed as a potential conflict of interest.
